# The Role of Water as a Reservoir for Antibiotic-Resistant Bacteria

**DOI:** 10.3390/antibiotics14080763

**Published:** 2025-07-29

**Authors:** Sameh Meradji, Nosiba S. Basher, Asma Sassi, Nasir Adam Ibrahim, Takfarinas Idres, Abdelaziz Touati

**Affiliations:** 1 Faculty of Nature and Life Sciences, Mohamed-Cherif Messaadia University, Souk Ahras 41000, Algeria; s.meradji@univ-soukahras.dz; 2 Department of Biology, College of Science, Imam Mohammad Ibn Saud Islamic University (IMSIU), Riyadh 13318, Saudi Arabia; nsbasher@imamu.edu.sa (N.S.B.); naabdalneim@imamu.edu.sa (N.A.I.); 3 Laboratory of Microbiology and Molecular Biology, Faculty of Sciences, Badji Mokhtar University, Annaba 23000, Algeria; asma.sassi@univ-annaba.dz; 4 Laboratory for Livestock Animal Production and Health Research, Rabie Bouchama National Veterinary School of Algiers, Issad ABBAS Street, BP 161 Oued Smar, Algiers 16000, Algeria; 5 Laboratoire d’Ecologie Microbienne, Faculty of Nature and Life Sciences, Université de Bejaia, Bejaia 06000, Algeria; abdelaziz.touati@univ-bejaia.dz

**Keywords:** antibiotic resistance genes, antibiotic-resistant bacteria, spread mechanisms, environmental pathways of transmission, waterborne pathogens

## Abstract

Water systems serve as multifaceted environmental pools for antibiotic-resistant bacteria (ARB) and resistance genes (ARGs), influencing human, animal, and ecosystem health. This review synthesizes current understanding of how antibiotics, ARB, and ARGs enter surface, ground, and drinking waters via wastewater discharge, agricultural runoff, hospital effluents, and urban stormwater. We highlight key mechanisms of biofilm formation, horizontal gene transfer, and co-selection by chemical stressors that facilitate persistence and spread. Case studies illustrate widespread detection of clinically meaningful ARB (e.g., *Escherichia coli*, *Pseudomonas aeruginosa*, *Klebsiella pneumoniae*) and mobile ARGs (e.g., *sul1/2*, *tet*, *bla* variants) in treated effluents, recycled water, and irrigation return flows. The interplay between treatment inefficiencies and environmental processes underscores the need for advanced treatment technologies, integrated monitoring, and policy interventions. Addressing these challenges is critical to curbing the environmental dissemination of resistance and protecting human and ecosystem health.

## 1. Introduction

Water, one of the most critical environmental reservoirs, acts as both a direct habitat and transmission route for antibiotic-resistant bacteria [1,2]. Water bodies such as rivers, lakes, oceans, and groundwater are susceptible to contamination with antibiotic residues, bacteria, and resistance genes through multiple sources [3,4]. These sources include agricultural runoff from livestock farming, untreated human waste, pharmaceutical industries, and wastewater treatment plants that often fail to remove all pharmaceutical contaminants.

Antibiotic residues in water create selective pressure, fostering the survival and proliferation of resistant strains of bacteria [5,6]. For instance, bacteria like *Escherichia coli* [7,8], *Salmonella* spp. [9,10], and *Pseudomonas aeruginosa* [9], which are typically found in wastewater or contaminated water sources, can harbor bacteria resistant to multiple antibiotics, such as β-lactams and tetracyclines [10]. These resistant bacteria can be transmitted to humans and animals through contaminated drinking water [11], recreational water activities, or by consuming contaminated food [12], such as fish or vegetables irrigated with polluted water [13,14].

Research employing traditional culture-based methods has shown that resistant strains can persist in water for extended periods, challenging eradication efforts. For example, a study by Zhang et al. reported the presence of over 150 distinct resistance genes in river water samples taken from urban and rural locations. Culture-independent methods, like metagenomics, have revealed a broader diversity of resistant bacteria and their genes within aquatic environments, offering a clearer picture of how water ecosystems harbor and propagate antimicrobial resistance (AMR) [15].

Waterborne diseases remain a key concern in developing regions where water treatment facilities may be inadequate, underscoring the need for better management practices. Addressing antibiotic pollution in water bodies, such as improved wastewater treatment and restrictions on pharmaceutical discharge, is vital in curbing the spread of antibiotic-resistant bacteria (ARB) [16].

**Figure 1:**
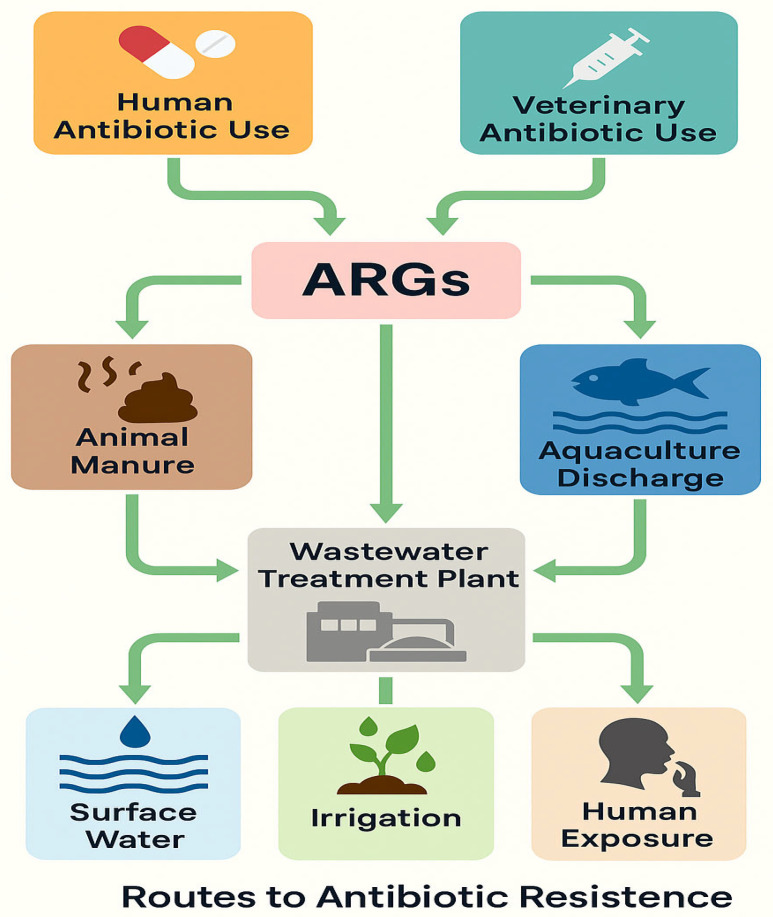
Environmental, clinical, and agricultural sources contribute to the circulation of ARGs.

Water plays a significant role as a reservoir for ARB, contributing to the spread and persistence of these pathogens in the environment (Figure 1). The presence of ARB in water bodies such as rivers, lakes, wastewater, and drinking water poses a serious public health threat [17] due to the potential for human exposure [18].

### Historical and Anthropogenic Drivers of Antibiotic Resistance and Environmental Dissemination

Antibiotic resistance genes (ARGs) have deep evolutionary roots. Functional metagenomic analyses have revealed that resistance mechanisms to major antibiotic classes including β-lactams, tetracyclines, and glycopeptides exist in microbial communities long before the advent of human antibiotic use. ARGs have been identified in 30,000-year-old permafrost sediments, demonstrating that these genes originally served ecological functions unrelated to medical or veterinary contexts. Horizontal gene transfer (HGT) later facilitated their spread across diverse bacterial populations [17,19,20].

In the modern era, the widespread and unregulated use of antibiotics in human and animal sectors has accelerated the dissemination of ARGs. A major contributor is the agricultural sector. In livestock systems, notably poultry, swine, and cattle, antibiotics such as tetracyclines, macrolides, sulfonamides, and β-lactams are routinely used for both treatment and growth promotion. This continuous selective pressure favors resistant bacteria such as *Escherichia coli*, *Salmonella* spp., and *Enterococcus* spp. These are shed in manure, which is commonly applied to agricultural fields, leading to contamination of soil, crops, and nearby water bodies. Genes like *blaCTX-M*, *mcr-1*, and *tet(M)* have been recovered from both livestock waste and human clinical isolates, demonstrating direct zoonotic and environmental transfer [21].

In aquaculture, antibiotics such as oxytetracycline, florfenicol, and sulfadimethoxine are used to prevent bacterial infections in fish and shrimp. These drugs persist in aquatic environments, selecting for ARGs like *tet*, *sul*, and *qnr*, which can accumulate in sediments and be transferred to native or opportunistic bacteria. Pathogens such as *Vibrio* and *Aeromonas*, commonly found in aquaculture settings, have been shown to harbor these ARGs, creating potential routes for resistance transmission to humans via seafood or contact with contaminated water [22,23].

Plant agriculture also contributes to ARG dissemination. In the United States, China, and other countries, antibiotics such as streptomycin and oxytetracycline are used to manage bacterial diseases in crops like apples, pears, and citrus. These treatments have led to the emergence of resistant plant pathogens such as *Erwinia amylovora*. Resistance genes including *strA* and *strB* have also been identified in non-pathogenic, plant-associated bacteria like *Pseudomonas* spp. and *Xanthomonas* spp., which can potentially transfer ARGs to human pathogens via plasmids and integrons [24,25].

The World Health Organization has highlighted the severity of the antimicrobial resistance crisis through its global priority pathogen list, which includes critical threats such as carbapenem-resistant *Acinetobacter baumannii*, *Pseudomonas aeruginosa*, and *Enterobacteriaceae*. These organisms are increasingly found outside clinical settings, including in agricultural and aquatic environments, where antibiotic residues and ARGs create ideal conditions for their selection and spread [26].

Collectively, these examples from livestock, aquaculture, and crop production underscore how anthropogenic use of antibiotics in non-clinical settings contributes to the environmental resistome. ARGs from these systems can ultimately reach humans through interconnected pathways including food consumption, direct contact, and contaminated water, reinforcing the importance of a “One Health” approach to antimicrobial resistance containment.

## 2. Sources of Antibiotic-Resistant Bacteria in Water

One health issue influencing life across humans, animals, and the environment is antibiotic resistance, which is found in one of the most basic resources for life water [27]. Antibiotic-resistant pathogens and their genes have been found in streams, lakes [28], rivers [29], and oceans [30]. They can often be traced back to hospital discharge [31], farms, or sewage systems (Table 1) [32]. Even properly functioning wastewater treatment systems may not fully remove resistant pathogens and their genes [33].

ARB in water environments originates from various sources, including wastewater treatment plants [34], agricultural runoff [11], urban stormwater [35], and landfill leachates [36]. Additionally, it can include human and animal excreta too [37]. This occurrence is primarily because the bacteria in these wastes are often exposed to antibiotics, whether for treatment, prophylaxis, or as growth promoters in livestock farming [33].

**Table 1:** Comparative summary of predominant ARGs, associated pathogens, regions, and detection methods by water source.

Water Source	Main ARGs	Associated Pathogens	Regions	Detection Methods	References
Wastewater Treatment Plants	*sul1,tet*, *blaCTX-M*	*E.coli*, *K. pneumoniae*, *P. aeruginosa*	Europe, Asia, Africa	qPCR, metagenomics	[34,38]
Hospital Effluents	*blaNDM*, *blaOXA-48*, *mcr-1*	ESKAPE pathogens, *Enterococcus* spp.	Middle East, Africa	Culture, PCR, WGS	[39,40]
Agricultural Runoff	*tetM*, *sul1*, *ermB*	*E. coli*, *Enterococcus* spp., *Salmonella* spp.	N. America, Asia	qPCR, metagenomics	[41,42]
Urban Stormwater	*sul1*, *tetA*, *blaTEM*	*E. coli*, *S. aureus*	USA, China	qPCR, MST	[43,44]
Drinking Water	*sul1*, *tetA*, *blaTEM*	*P. aeruginosa*, *Legionella* spp.	Global	Culture, qPCR	[45,46]
Recycled Water	*blaCTX-M*, *sul1*, *qnrS*	*E. coli*, *P. aeruginosa*	Europe, Australia	qPCR, culture	[47,48]

### 2.1. Wastewater Treatment Plants (WWTPs)

WWTPs are complex microbial environments with a mix of pathogenic and environmental bacteria. They constitute reservoirs for ARBs and antibiotic resistance genes (ARGs). While designed to treat sewage, conventional treatment processes often fail to eliminate ARBs and ARGs, releasing them into receiving water bodies. Studies show that WWTPs are both points of antibiotic accumulation and hotspots for ARBs and ARGs [38].

Since antibiotics are not completely broken down in the body, they are excreted via urine and feces, eventually reaching wastewater treatment plants (WWTPs) [49]. If these plants do not effectively remove the contaminants, antibiotics and resistant bacteria may be released into freshwater systems like rivers and lakes [50]. Biological treatment systems like activated sludge may inadvertently promote horizontal gene transfer under antibiotic-selective pressures [51]. Moreover, standard disinfection processes (e.g., chlorination, UV) are not fully effective at degrading extracellular DNA, allowing ARGs to persist in effluents. Advanced technologies such as ozonation, membrane bioreactors, and UV/H_2_O_2_ oxidation achieve higher ARG removal efficiencies, but their adoption is limited by cost, energy needs, and maintenance complexity, particularly in LMIC settings [51].

For example, Okoh et al. studied WWTPs in the Eastern Cape and highlighted their role as significant pollution sources for pathogens and antimicrobial resistance genes [36]. These results indicated that WWTPs in the Eastern Cape were identified as important sources of antimicrobial-resistant *E. coli*. They found that 32.7% of *E. coli* isolates exhibited multidrug resistance to 15 antibiotics, with resistance genes like *mcr-1* and *ermA* detected in effluent samples [52].

This finding is supported by several studies from developing and developed regions [35,36], which discussed the spread of antimicrobial resistance due to inadequate removal during treatment processes. Bacteria identified in this study include *P. aeruginosa*, *Klebsiella pneumoniae*, and various *Enterococcus* spp. The study conducted by Yang et al. took place in a large-scale municipal wastewater treatment plant located in Chuzhou, China. The researchers used Illumina MiSeq sequencing to analyze the structure of microbial communities in the activated sludge of this facility. The results revealed that the dominant phyla were Proteobacteria, Bacteroidetes, and Actinobacteria, with a notable presence of Firmicutes and Chloroflexi [53].

Additionally, research shows that the coexistence of pharmaceuticals and personal care products (PPCPs), biocides, metals, and agrochemicals in these environments further contributes to the development of antibiotic resistance among bacterial populations [54]. In many WWTPs, the combined presence of these chemicals creates significant evolutionary pressure, thereby promoting the selection and propagation of resistant bacteria. In South-eastern China, a study on animal farms found 22 ARGs conferring resistance to major antibiotics, including tetracyclines, sulfonamides, quinolones, aminoglycosides, and macrolides. The spread of these ARGs was dominated by *sul* genes, followed by *tet* and *erm* genes. The presence of metals like copper, arsenic, and zinc in agricultural settings may trigger co-selection for these ARGs, enhancing their persistence in the environment [55]. A study conducted in Tunisia, on sewage sludge-amended soils, revealed varying indices of antibiotic resistance, with the highest values observed for amoxicillin (AMX), doxycycline (DOX), and azithromycin (AZI). The prevalence of these resistant bacteria was influenced by factors such as antibiotic consumption patterns, wastewater treatment processes, and the storage conditions of sewage sludge [56]. A systematic review analyzed the impact of point sources such as wastewater treatment facilities and agricultural operations on the prevalence of ARB in natural environments. The study highlighted the need for improved study designs and analytical methods to quantify the effect of these point sources on ARB dissemination [57]. While WWTPs represent centralized, high-load entry points, agricultural runoff introduces diffused but sustained ARG pressure into surface and groundwaters. This transition from point to non-point sources highlights the synergistic role of farming practices in amplifying environmental resistance.

### 2.2. Agricultural Runoff

The extensive use of antibiotics in agriculture, particularly in livestock farming, contributes to the presence of ARB in water systems. When used as fertilizer or improperly managed, manure from treated animals can introduce ARB and ARGs into surface and groundwater through runoff [58]. Runoff during rainfall can transport these resistant elements into nearly all water bodies, increasing the environmental load of ARB.

Recent studies have underscored that agricultural runoff is a significant pathway for disseminating antimicrobial-resistant bacteria and resistance genes into the environment. For instance, Zhu et al. demonstrated that animal manure, especially from swine farms, is a reservoir of diverse and abundant antibiotic resistance genes, which can be mobilized into water systems through runoff [41]. The research was conducted in China, targeting three major swine-producing regions: Beijing, Jiaxing, and Pu Tian. These farms collectively housed over 10,000 pigs annually, representing intensive farming practices typical in China. The study identified 149 ARGs across manure samples, with resistance mechanisms spanning multiple antibiotic classes, including tetracyclines, sulfonamides, β-lactams, and macrolides [41]. Heuer et al. focused on EU countries (Germany, the Netherlands), where manure application increased *tet* and *sul* genes in agricultural soils. Dairy cow manure was a major reservoir of *erm* genes [42]. Mobile genetic elements (plasmids, transposons) facilitated ARG exchange between manure-borne *E. coli* and soil bacteria like *Pseudomonas* spp. [59]. Co-selection via heavy metals (copper, zinc) in manure amplified ARG persistence.

Complementing these findings, Guo et al. provided evidence of seasonal and spatial variability in the abundance of resistance genes within agricultural watersheds, highlighting that runoff events can sustain elevated levels of these genes in surface waters [60]. Similarly, Cheng et al. found that rural watersheds receiving agricultural runoff contain multiple resistance genes, directly linking these findings to farming practices, particularly the widespread use of antibiotics in livestock production [61]. This finding emphasizes the critical need for improved management practices to mitigate the environmental and public health risks associated with the spread of antibiotic resistance from agricultural sources.

### 2.3. Urban Stormwater

Urban runoff is a notable pathway for the dissemination of ARB and ARGs into aquatic environments. Factors such as combined sewer overflows, improper medication disposal, and surface runoff contribute to the presence of these contaminants in urban waterways [62]. Several studies have characterized its role in spreading ARB into downstream water bodies, highlighting the multifaceted nature of resistance dissemination.

For example, O’Malley et al. reviewed how urban stormwater acts as a hotspot for ARGs due to the convergence of pollutants, mobile genetic elements, and selective pressures such as heavy metals. Their work synthesized data from 12 separate storm–sewer outfalls in the Tampa Bay metropolitan area of Florida, USA. In that Tampa Bay study, the sulfonamide resistance gene *sul1* and the tetracycline resistance gene *tetA* were highly prevalent, being detected in over 95% of all grab-sampled stormwater outfalls, which emphasizes that traditional gray stormwater infrastructure may inadvertently facilitate the transport of these resistance elements into recreational and drinking water sources [62]. While the review aggregates global data, specific studies in Milwaukee highlight localized impacts, such as ARG transport into Lake Michigan [63]. The confluence of MGEs (e.g., plasmids, integrons) and contaminants in stormwater creates a reservoir for horizontal gene transfer, posing risks to public health through recreational or drinking water exposure [62]. Additionally, O’Malley et al. distinguished between intracellular and extracellular ARGs in stormwater, revealing distinct seasonal and spatial patterns that further complicate the overall resistome profile of urban runoff [64].

Similarly, Lee et al. profiled four municipal separate storm–sewer (MS4) outfalls during spring–summer 2017 in Columbus, OH, USA, and showed that wet-weather (storm) flows discharged much higher densities of *E. coli* than baseflow; microbial-source tracking (MST) found ruminant fecal markers in 91% of samples. Metagenomic analysis revealed a resistome dominated by sulfonamide resistance genes (*sul1*, *sul2*), which were strongly associated with urban and agricultural runoff, as well as tetracycline resistance genes (*tetA*, *tetC*, *tet(O)*, *tet(W)*), which correlated with fecal indicators like *E. coli*. Beta-lactam resistance genes such as *bla*_TEM_ (indicative of resistance to penicillin-type antibiotics) and macrolide resistance genes like *ermF* (frequently found in extracellular DNA) were also widespread. These ARGs were often co-located with bacterial pathogens, including *Staphylococcus aureus* and *Salmonella enterica Typhimurium*, highlighting risks of co-transfer between resistance and pathogenicity traits [43].

In another study, stormwater runoff in humid Chinese cities contained 1.8× higher micropollutant concentrations (antibiotics, endocrine disruptors) during wet weather compared to dry conditions. ARGs such as *sul1* and *tetC* were prevalent, with stormwater discharge contributing significantly to surface water contamination, particularly in areas with combined sewer overflows [65].

Di Cesare et al. evaluated the impact of a moderate rain event (4 mm/h) on ARG levels in a large subalpine river in the Italian Alps. They used qPCR to track five ARGs—*tetA* (tetracycline), *ermB* (macrolide), bla_CTX-M_ (β-lactam), *sulII* (sulfonamide), and *qnrS* (quinolone)—and found that total ARG abundance increased 24-fold during the rain compared to the annual average. This spike coincided with higher concentrations of total phosphorus, ammonium-N, and microbial aggregates, implicating overland runoff and catchment sources in ARG mobilization [66].

Ahmed et al. were the first to link sewage-associated marker genes with ARGs in an Australian urban river, sampling untreated sewage influent and 12 sites along the Brisbane River and its tributaries under baseflow and stormflow. They quantified fecal indicators (*E. coli*, enterococci), sewage markers (e.g., HF183), and ARGs such as *sul1*, *intI1*, *tetW*, and *ermF*. Stormflow samples showed markedly higher abundances of both sewage markers and ARGs compared to dry weather, with strong correlations (r > 0.8) between HF183 and multiple ARGs, highlighting rain-driven mobilization of human-derived resistance elements [67]. In contrast to the broader environmental contributions, hospital effluents introduce highly concentrated loads of multidrug-resistant pathogens and clinically relevant ARGs into wastewater networks.

### 2.4. Hospital Effluents

Due to medical treatments and laboratory activities, wastewater from healthcare facilities often contains high concentrations of antibiotics and ARB. If not adequately treated before discharge, these effluents can introduce resistant bacteria into municipal sewage systems and natural water bodies [68].

Hospital wastewater is a recognized hotspot for antibiotic-resistant bacteria due to the heavy use of antibiotics in clinical settings. Multiple studies have shown that hospital effluents contain high levels of antibiotic residues and resistant pathogens. For example, a study conducted in Egypt identified MDR bacteria in hospital WWTP effluents, with isolates including *Staphylococcus haemolyticus*, *Enterococcus faecalis*, and *E. coli* being recovered via 16S rRNA gene sequencing. These isolates resisted standard antibiotics such as tetracycline, ampicillin, and ceftazidime at concentrations up to 25 ppm, although they were susceptible at higher concentrations [39].

Another study from Ethiopia focused on the bacteriological quality of hospital wastewater and found that the most frequently isolated pathogens were *S. aureus*, *E. coli*, *Citrobacter* spp., and *Acinetobacter* spp. [69]. Notably, *K. pneumoniae* and *P. aeruginosa* have also been reported in hospital effluents, critical ESKAPE pathogens responsible for hospital-acquired infections. For instance, research from Benin documented a predominance of non-enterobacteria and *Acinetobacter* spp. in hospital effluents, highlighting their potential to disseminate resistance genes into the environment [70]. In addition, recent metagenomic approaches have revealed that hospital effluents are reservoirs for clinically significant resistance determinants, including extended-spectrum beta-lactamases (ESBLs) and carbapenemases [71]. As treated wastewater is increasingly reused or discharged into potable water sources, understanding how ARB and ARGs persist or are inadequately removed during advanced water treatment becomes crucial.

### 2.5. Drinking Water Treatment and Distribution Systems (DWDS)

Even after treatment, drinking water can harbor ARB and ARGs. Factors such as biofilm formation within distribution systems and using disinfectants like chlorine can influence the prevalence and persistence of these contaminants in potable water [72]. Another review discussed how conventional drinking water treatment processes might not effectively eliminate ARB, potentially extending antibiotic resistance and posing risks to human health. The authors emphasized the importance of understanding the frequency and quantity of bacteria with antibiotic-resistant mutations in drinking water systems [73].

DWDS are critical for ensuring safe water; however, several investigations have detected low levels of ARB and ARGs in both finished drinking water and tap water, even after conventional treatment processes such as coagulation, sedimentation, filtration, and disinfection.

For instance, Xi et al. examined drinking water treatment plants in Michigan and Ohio using culture-based and molecular methods. Their work revealed that although the overall bacterial load decreased significantly during treatment, the relative abundance of ARGs increased in tap water compared to source water. The study identified a range of ARGs associated with resistance to beta-lactams, aminoglycosides, tetracyclines, and fluoroquinolones. Moreover, regrowth of heterotrophic bacteria in the distribution system was observed, with species such as *Pseudomonas* spp. and *Acinetobacter* spp. emerging as a notable ARB [46].

Another study by Sanganyado and Gwenzi reviewed ARB’s occurrence, removal, and human health risks in drinking water systems. Their review provides a global synthesis of ARB and antibiotic resistance genes ARGs in drinking water systems, highlighting case studies from China, sub-Saharan Africa, Europe, and North America and emphasizing both conventional and advanced treatment processes. The most commonly detected ARGs across these systems include sulfonamide (*sul1*, *sul2*), tetracycline (*tetA*, *tetM*), β-lactam (*ampC*, *bla*_TEM_), macrolide (*ermB*), and the class 1 integron integrase gene (*intI1*), with opportunistic pathogens such as *P. aeruginosa*, *Legionella pneumophila*, non-tuberculous mycobacteria, and ESBL-producing *E. coli* frequently persisting throughout treatment. This work underscored that conventional disinfection (e.g., chlorination) may not effectively remove ARGs and, in some cases, may even select for resistant populations [45]. Similarly, a study by Chen et al. conducted targeted qPCR and 16S rRNA sequencing on bulk water and biofilm samples from a full-scale DWTP and distribution system in Shanghai, China, using Yangtze River source water. They quantified six key markers, *sul1*, *sul2*, *tetA*, *tetM*, *ampC*, and *intI1*, and found that although absolute ARG abundances dropped post-treatment, relative abundances in biofilms remained high. Opportunistic pathogens detected by qPCR included *Mycobacteria* spp., *Legionella* spp., *L. pneumophila*, *P. aeruginosa*, and *Hartmannella vermiformis*, all of which correlated strongly with ARG levels, underscoring biofilms as reservoirs for ARG dissemination [74].

These studies have highlighted that while treatment processes effectively reduce overall microbial counts, the selective pressures exerted during treatment and within distribution pipelines may foster the proliferation of ARB. Notably, ARB pathogens, such as *E. coli*, *P. aeruginosa*, and *Acinetobacter baumannii*, that can cause infections in vulnerable populations have been identified in tap water. In some instances, the levels of ARGs related to multidrug resistance and specific antibiotic classes (e.g., beta-lactams) were higher in distributed water than in finished water directly from treatment plants.

### 2.6. Recycled Water

Reusing treated wastewater for purposes like irrigation can be a source of ARB exposure. Studies have detected various resistant bacteria in recycled water, highlighting the need for comprehensive risk assessments and effective treatment strategies to mitigate potential health risks [47].

Recycled water, which is increasingly used for irrigation and industrial applications, has been shown to harbor ARB and ARGs [75,76]. Several studies have demonstrated that even after treatment, recycled wastewater may retain or even select for resistant bacteria due to suboptimal removal of antibiotics and the persistence of biofilms [77].

For example, a study by Chen et al. detected a diverse array of ARGs in recycled wastewater used for agricultural irrigation. In this work, researchers identified clinically relevant bacteria, including multidrug-resistant *E. coli*, *E. faecalis*, and *P. aeruginosa*, in samples collected from recycled water systems [78]. Similarly, Drigo et al. reported the effectiveness of recycled water treatment plants in eliminating human opportunistic pathogens and antimicrobial-resistant microorganisms. The study identified several enteric opportunistic pathogens, including *A. baumannii*, *E. coli*, *E. faecalis*, *K. pneumoniae*, and *P. aeruginosa*. The findings indicated that while the treatment plants removed over 95% of these pathogens and resistance genes, some subpopulations survived disinfection and had the potential to regrow within the recycled water systems. This suggests that despite high-quality treatment, certain antibiotic-resistant bacteria may persist in recycled water, posing potential risks if the water is reused [48].

### 2.7. Irrigation and Non-Potable Uses

Recycled, non-potable water used for irrigation can be a significant source of ARB that may be transferred to crops and, ultimately, to humans. Several studies have investigated this issue.

A study by Odonkor and Addo examined water sources used for irrigation in rural settings in Ghana and reported that surface water sources, particularly dams, exhibited high *E. coli* contamination (up to 3.22 × 10⁵ CFU/100 mL) and multidrug resistance (49.48% of isolates). Resistance to penicillin (32.99%) and tetracycline (21.45%) was common, with *sul1* and *tet* genes frequently detected. Their findings highlighted that even water not intended for direct human consumption can serve as a reservoir for ARB, raising concerns about foodborne exposure [79]. Similarly, Larson et al. conducted research in Peru and detected multidrug-resistant *E. coli* and *Klebsiella* spp. on vegetables irrigated with reclaimed water, suggesting that recycled water used in agriculture may facilitate the transfer of resistance genes onto produce [80]. In addition, research in Nigeria has identified antibiotic-resistant *Enterococcus* spp. and *Salmonella* spp. in water sources used for vegetable irrigation, highlighting risks to food safety and public health. Key studies focus on regions such as Lagos State and Northwest Nigeria, where agricultural runoff, untreated wastewater, and livestock activities contribute to contamination of irrigation water with ARB [81].

In Idaho, USA (Snake River Watershed), irrigation return flows (IRFs) contained *E. coli* and *enterococci* with resistance to tetracycline (13%) and macrolides (*ermF*). MDR patterns were observed in 12% of isolates, linked to agricultural runoff and livestock activities [82].

The presence of foodborne pathogens in irrigation water is common. These organisms could remain viable and survive for a very long time and develop antibiotic resistance, posing a great threat to public health. Pathotypes of *E. coli* and *Enterococcus* spp. are microorganisms that survive under these conditions [83]. *Salmonella* spp. can also be found in the agricultural environment and is one of the most common causes of severe foodborne diseases.

Among the diverse sources contributing to ARG dissemination in aquatic environments, hospital effluents are consistently reported to have the highest concentrations of clinically significant resistance genes, such as *blaNDM*, *blaOXA-48*, and *mcr-1* [40]. Though volumetrically smaller than agricultural runoff, these effluents are enriched in multidrug-resistant pathogens and last-resort resistance genes. In contrast, agricultural runoff, particularly from intensive livestock operations, contributes a broader volume of ARGs such as *tet*, *sul*, and *erm* due to the scale of antibiotic application in animal production [42]. Urban stormwater runoff has also emerged as a significant source, containing mixtures of human and animal waste, pharmaceuticals, and metals, all of which drive co-selection [51]. Treated recycled water may harbor lower ARG levels but still poses a risk of ARG persistence, especially where advanced treatments are lacking.

### 2.8. Regional Case Study

#### 2.8.1. Antibiotic Resistance in North African Water Bodies

In a recent survey, qPCR and metagenomic surveys across North Africa have revealed that sulfonamide (*sul1*), tetracycline (*tetM*), and extended-spectrum β-lactamase (*bla*_CTX-M_) genes are not only widespread but also often highly abundant in both the water column and underlying sediments of coastal lagoons, estuaries, and river systems. Tunisia (Bizerte Lagoon): In sediment cores and surface waters of this heavily urbanized Mediterranean lagoon, *sul1* copy numbers reached (sediment) and (water), with *tetM* and *blaCTX-M* detected at similarly elevated levels [84]. In Morocco (Oued Sebou Estuary): Surveys here found *sul1* and *tetM* in 100% of sediment–water-paired samples, with *blaCTX-M-15* present in over 60% of isolates, highlighting the impact of upstream agricultural runoff and untreated urban discharges [85]. *Vibrio alginolyticus* isolated from sea bream in Egyptian aquaculture farms near Port Said (Nile Delta) harbored *sul1* (28.8% of isolates), *tetA*, and *blaTEM* genes. This study underscores marine ecosystems’ role as AMR reservoirs, with multidrug-resistant bacteria likely spreading to coastal waters through aquaculture effluents [86]. Recent investigations of Algerian water systems have revealed the presence and environmental persistence of clinically important β-lactamase and colistin resistance determinants. A broader survey in Batna targeting hospital tap water, sewage, and environmental effluents reported a high level of resistance in both tap water and wastewater. The most commonly found carbapenem resistance mechanism was the *OXA-48* enzyme, but other carbapenemases were also detected. In addition, the *mcr-1* gene was detected in 18 *E. coli* of different sequence types [87]. In addition, along the Soummam River near Béjaïa, screening of 12 river water samples recovered 20 carbapenem-resistant *Enterobacteriaceae* isolates, 17 of which carried *blaOXA-48*-like and 3 harbored *blaOXA-244* genes [88].

Together, these studies underscore that both freshwater and coastal systems in Algeria act as reservoirs for ESBLs, MBLs, carbapenemases, and plasmid-mediated colistin resistance, highlighting the urgent need for reinforced wastewater treatment and continuous ARG surveillance to mitigate environmental and public health risks.

#### 2.8.2. Antibiotic Resistance in European Water Bodies

A comprehensive survey across ten European countries analyzed effluents from 16 wastewater treatment plants (WWTPs) and their receiving rivers. Quantitative PCR (qPCR) detected antibiotic resistance genes (ARGs) such as *sul1*, *intI1*, and *blaOXA-58* in all samples. The study found that the abundance of these genes was inversely correlated with the number of biological treatment steps implemented at the WWTPs, suggesting that advanced wastewater treatment processes play a key role in mitigating ARG dissemination into the environment [89].

#### 2.8.3. Southern Germany: River Biofilms and Sediments

A targeted study of a river influenced by a small WWTP in Southern Germany examined surface water, sediment, and river biofilms. qPCR analyses revealed the presence of *blaTEM*, *ermB*, *tetM*, and *sul1*. ARG concentrations were significantly higher downstream of the WWTP, indicating that treated effluent discharge is a major contributor to ARG propagation in fluvial ecosystems [90].

#### 2.8.4. Italy: Maiella National Park

Even in protected areas like Maiella National Park in Italy, ARGs were prevalent. Multidrug-resistant bacteria were isolated from water and fecal samples of both livestock and wildlife. Detected resistance genes included *mcr-4*, *blaOXA-48*, *blaTEM*, and *tetB*. This underscores the pervasiveness of ARGs even in relatively pristine environments, highlighting the role of human and animal activities in spreading resistance determinants into natural ecosystems [91].

#### 2.8.5. Antibiotic Resistance in Asian Water Bodies

In Asia, aquatic environments are increasingly recognized as significant reservoirs of antibiotic resistance genes (ARGs), driven by human activity and aquaculture. In South Korea, a study conducted in South Jeolla Province and Jeju Island assessed effluents from coastal aquaculture farms. Quantitative PCR (qPCR) analysis detected 22 distinct ARGs, with *tetB* and *tetD* being the most prevalent. These resistance genes were strongly associated with bacterial genera such as *Vibrio* spp. and *Marinomonas* spp., underscoring the role of aquaculture effluents in facilitating the spread of ARGs in marine ecosystems [92]. Similarly, investigations in Xiamen City, China, revealed widespread ARG contamination in lagoon waters impacted by urban stormwater runoff. Using high-throughput qPCR and metagenomic techniques, researchers identified 285 ARGs, including *sul1*, *tetO*, and *qnrS*, indicating that rapid urbanization and insufficient wastewater management are key contributors to ARG dissemination in Chinese aquatic systems [44]. These findings highlight how both aquaculture and urban development are critical drivers of environmental antibiotic resistance in Asia.

Interpreting the environmental burden of ARGs requires careful evaluation of detection methods. The following section critically examines the methodological strengths and limitations that influence how resistance is quantified across water systems.

## 3. Methodological Considerations

Understanding the strengths and limitations of various ARG detection methods is essential for interpreting findings across environmental studies. The primary approaches include culture-based isolation, quantitative PCR (qPCR), and metagenomics, each with distinct biases and constraints.

Culture-based methods allow isolation of live bacterial strains for phenotypic resistance profiling and downstream molecular analyses. However, they capture only a small fraction of environmental microbial diversity, typically <1% of total bacteria, and fail to detect viable but non-culturable (VBNC) organisms or extracellular ARGs (eARGs) [93]. Their use can thus lead to significant underestimation of the resistome in aquatic environments.

Quantitative PCR (qPCR) offers high sensitivity and specificity for detecting known ARGs in complex environmental samples [94]. It is widely used for quantifying genes such as *sul1*, *intI1*, *blaTEM*, and *tetA* in wastewater and surface water [95]. However, qPCR is inherently limited to predefined targets and is vulnerable to primer-template mismatches, amplification biases, and the inability to distinguish intracellular ARGs from free extracellular DNA [96]. This can lead to the overestimation of the abundance of functional resistance if eARGs persist post-treatment [97].

Shotgun metagenomics enables untargeted, high-resolution profiling of entire microbial communities and their associated resistomes [98]. It is especially valuable for identifying novel or rare ARGs as well as co-occurring mobile genetic elements (e.g., integrons, plasmids). However, accurate quantification depends on sequencing depth, bioinformatic pipeline choices, and the completeness of reference databases like CARD, ResFinder, or ARGs-OAP [99]. Misannotation or false positives can arise when ARG homologs are misclassified.

**Figure 2:**
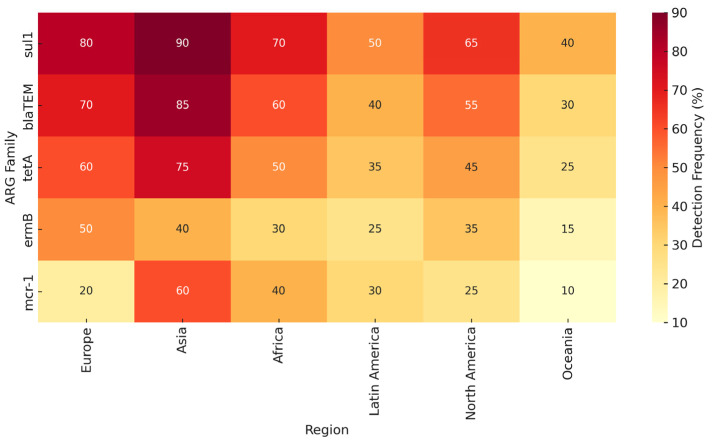
Heat-map-style diagram showing ARG prevalence across continents and detection methods (culture, qPCR, metagenomics).

In summary, no single method provides a complete picture of environmental AMR. Combining multiple approaches or interpreting results within their methodological context is essential. This triangulation is reflected in our comparative synthesis (Table 2) and global detection map (Figure 2), which integrate findings across platforms and regions.

Given the variability in methods and environmental contexts, synthesizing ARG prevalence across regions and water sources provides a clearer understanding of global resistance patterns.

The presence of ARB in water systems is not solely dependent on source inputs but also on microbial and physicochemical mechanisms that promote persistence and spread. We now shift focus to key ecological and molecular drivers such as biofilm formation, and microplastic-mediated co-selection.

## 4. Mechanisms of Persistence and Spread in Water

### 4.1. Biofilm Formation in Natural and Engineered Systems

Bacteria readily form biofilms on surfaces such as sediments, pipes, and microplastics in aquatic environments. These biofilms provide a protective environment where bacteria can survive adverse conditions, including disinfection, and maintain high cell densities. Biofilms act as reservoirs, periodically releasing ARB into water supplies through detachment, posing direct public health risks [100].

Biofilms in aquatic environments are indeed highly conducive to horizontal gene transfer (HGT) due to the dense microbial communities they support and the extracellular polymeric substances (EPS) matrix that enhances survival and communication [101]. This has been demonstrated in various studies focusing on both natural and engineered aquatic systems, including those involving microplastics, which serve as novel substrates for biofilm growth and ARG exchange [102,103]. This phenomenon is evident in drinking water distribution systems and natural water bodies [74]. HGT is further amplified in biofilms due to high cell density and proximity, allowing ARGs to transfer between species, including uptake of free DNA from dead cells by living bacteria, perpetuating resistance even after disinfection [74].

A study demonstrates that biofilms on plastic debris transport ARB/ARGs across oceans. A study found *mcr-1* in biofilms on polyethylene particles in the Mediterranean Sea. Biofilms on sediment particles act as ARG reservoirs. *E. faecalis* with *vanA* (vancomycin resistance) transfers genes to aquatic *Vibrio* spp., creating hybrid pathogens [104]. Biofilms in distribution systems release ARB (e.g., *L. pneumophila* with *ermB*) into tap water, especially in aging infrastructures. In addition, biofilms in trickling filters or membrane bioreactors inadvertently enrich ARGs (*bla*_NDM_, *sul1*) due to prolonged exposure to sub-inhibitory antibiotics [105].

Biofilms on root surfaces facilitate ARG exchange between soil bacteria and plant endophytes, enabling food chain contamination. In addition, biofilms accumulate metals (Zn, Cu) and disinfectants, co-selecting for resistance. For example, *czcA* (zinc resistance) and *mecA* (methicillin resistance) are linked on the same plasmid in *Staphylococcus* biofilms [103].

### 4.2. Microplastics as Novel Reservoirs and Vectors of ARGs

Microplastics have rapidly become recognized as a distinct ecological niche for antibiotic-resistant bacteria and genes. Their hydrophobic surfaces concentrate organic pollutants, heavy metals, and residual antibiotics, creating microzones of intense selective pressure. In the Danube River, Nguyen et al. found that microplastic-associated biofilms harbored ARG densities up to five times higher than surrounding suspended particles, with integron-bearing *Pseudomonas* spp. and *Aeromonas* spp. dominating the plastisphere community. Moreover, ocean currents can transport these colonized plastics thousands of kilometers, effectively shuttling resistance traits across biogeographic boundaries and connecting distant ecosystems in a single resistome network [104].

In addition, emerging research indicates bacteria colonizing these plastic particles benefit from a stable niche that can protect them from environmental stressors and promote gene exchange [105]. Studies have shown that biofilms on microplastics tend to be thicker and harbor higher densities of ARB compared to those on natural surfaces, potentially increasing the risk of ARG dissemination [106,107].

Research highlights the unique microbial communities found on microplastics, including pathogens such as *Vibrio* spp., *Pseudomonas* spp., and even human and fish pathogens, which tend to be more abundant in plastic biofilms than in natural environments. These biofilms also facilitate horizontal gene transfer (HGT), increasing the spread of ARGs within aquatic ecosystems [107,108].

Additionally, the movement and residence time of microplastics in water bodies shaped by hydrological and physical processes affect how microbial communities form and change on plastic surfaces, influencing ARG dissemination potential further [109].

### 4.3. Co-Selection by Emerging Chemical Stressors

Beyond antibiotics and metals, various emerging pollutants, including quaternary ammonium biocides, nanoparticle disinfectants, and pharmaceutical metabolites, can co-select for multidrug resistance. Bahded et al. demonstrated in laboratory experiments that exposure to sub-lethal concentrations of silver nanoparticles induces efflux pump overexpression in *A. baumannii*, which simultaneously confers cross-resistance to fluoroquinolones [110]. Field studies by Russo et al. linked high concentrations of benzalkonium chloride (a common surfactant) in Italian hospital stormwater to elevated class 1 integron prevalence. These findings underscore that controlling AMR in water requires a broad lens on all co-selective agents, and not just conventional antibiotics [111].

Other pollutants, such as heavy metals (e.g., mercury, arsenic) and disinfectant by-products, also contribute to selecting resistant bacteria. These substances can co-select for ARGs because resistance mechanisms (such as efflux pumps) can confer protection against multiple types of stressors [112]. Heavy metals in water, often from industrial discharge or pipe corrosion, further enrich the resistome by favoring bacteria harboring metal resistance genes and ARGs [113].

Co-selection, where non-antibiotic environmental stressors select for antibiotic resistance, is increasingly recognized as a major contributor to ARG persistence in water environments. Heavy metals such as copper, zinc, and mercury, as well as disinfectants like quaternary ammonium compounds (QACs), are known to exert selective pressures via shared resistance mechanisms. These include multidrug efflux pumps (e.g., *MexAB-OprM*), stress-response regulators (*SoxRS*, *MarA*), and co-located resistance genes on integrative conjugative elements or plasmids (e.g., *czcA*, *qacEΔ1*, *mecA*) [114,115]. Diverse ARGs associated with metals have been discovered, such as coupling of resistance genes for methicillin with Zn [116], tetracycline with Cu [117], and multiple antibiotics with Hg [118]. For instance, *Acinetobacter baumannii* exposed to silver nanoparticles developed fluoroquinolone resistance through efflux activation, while QAC exposure was linked to class 1 integron enrichment in hospital wastewater [119]. These mechanisms underscore the need to regulate both antibiotics and chemical co-selectors in wastewater discharge.

## 5. Public Health and Food Safety Impacts

ARB in water create a multifaceted threat to human health and food safety by acting as both infection sources and environmental reservoirs of resistance. Infected drinking or recreational water can introduce multidrug-resistant pathogens that evade standard treatments. For example, surveys of community water sources have found that 50% of *E. coli* isolates were MDR, and many had high multi-antibiotic resistance indices [79]. Larson et al. directly linked ARB in household drinking water to human carriage of resistant *E. coli*, highlighting water as a transmission route in rural communities [80]. Irrigation amplifies the problem: Weidhaas et al. detected multiple resistance gene families on vegetables irrigated with reclaimed wastewater, demonstrating that ARB on crops introduce resistance into the food chain [120]. Such findings implicate reclaimed or contaminated irrigation water in foodborne exposures to ARB. At the same time, natural and engineered water systems spread resistance more broadly: La Rosa et al. emphasized that wastewater treatment plants accumulate ARB/ARGs and then disseminate them via treated effluent, irrigation, and water reuse into agricultural lands and aquatic ecosystems [38]. Similarly, Fahrenfeld et al. observed that treated wastewater distribution often increases ARG diversity (via microbial regrowth), meaning end-use water (including for irrigation) can carry more resistance than the source [77]. Significantly, even disinfection steps can backfire: Sanganyado et al. noted that chlorination may promote horizontal transfer of resistance genes from environmental ARB to human pathogens in distribution systems [45]. These studies link ARB in water to elevated human infection risk and foodborne transmission, while also showing that water bodies and reuse practices serve as ecological reservoirs that spread resistance across human and environmental interfaces [33,120].

## 6. Environmental Transmission Pathways

**Figure 3:**
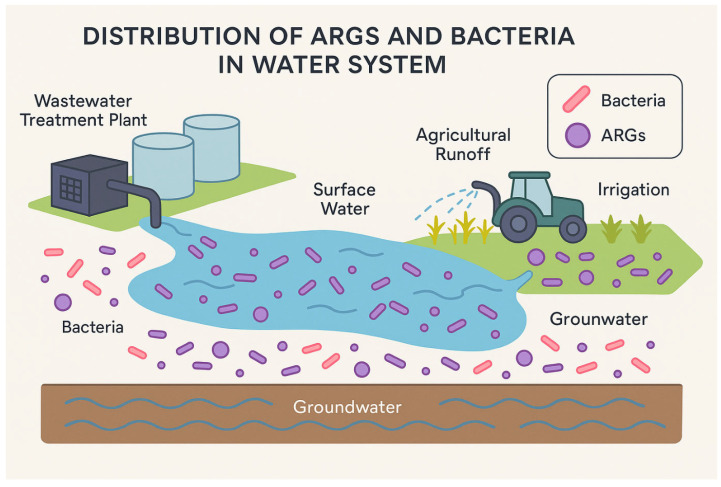
Schematic of potential AMR bacteria and ARGs transmission pathways in the water system.

Environmental pathways play a key role in the transmission and persistence of AMR across ecosystems, linking clinical settings with natural environments (Figure 3). Several studies have examined these pathways and identified various ARB that move between water, soil, wildlife, and the human gut. For example, Xi et al. demonstrated that drinking water treatment and distribution systems could serve as reservoirs for ARGs and ARB, with species such as *E. coli*, *P. aeruginosa*, and *A. baumannii* being detected even after treatment [46]. Similarly, studies in wastewater treatment plants have shown that hospital effluents and agricultural runoff contribute significantly to environmental AMR; research by Sanganyado et al. reviewed how ARB, such as *Enterococcus* spp. and *K. pneumoniae*, are disseminated via treated wastewater into natural water bodies [45].

Moreover, research investigating recycled water and irrigation has found that water reused for agricultural purposes can harbor multidrug-resistant *E. coli*, *Enterobacter* spp., and even *Salmonella* spp., suggesting that these water sources are key vectors for transferring resistance genes to crops and, ultimately, to humans [121]. In soil, antibiotic residues from manure and wastewater can select for resistant organisms, with studies revealing high levels of resistance in environmental isolates of *Pseudomonas* spp. and *Acinetobacter* spp. from agricultural lands [122]. Additional pathways include the persistence of ARGs in biofilms along water distribution systems and in sediments of rivers and lakes [123].

ARGs have been detected in biofilms from various freshwater ecosystems, including rivers and wetlands, indicating their role as reservoirs of resistance in aquatic environments [124].

AMR is no longer confined to clinical settings; it is now recognized as a global problem, with environmental pathways playing a crucial role in the dissemination and persistence of antibiotic-resistant bacteria and antibiotic resistance genes. A study conducted by Kusi et al. provided a comprehensive review of how surface waters become hotspots for AMR and examined the public health implications of these environmental reservoirs. The authors described several interconnected pathways through which antimicrobial resistance is developed and transmitted in aquatic environments [1]. Although numerous studies have documented the presence of ARGs in surface waters, sediments, and treated effluents, establishing a direct, causal relationship between environmental exposure and resistant infections in humans remains scientifically complex. This is primarily due to methodological challenges such as the absence of longitudinal cohort data, the inability to track specific mobile genetic elements from environmental to clinical isolates, and confounding exposure routes such as foodborne or zoonotic pathways [125,126]. For instance, metagenomic analyses in Ghana and Peru have shown overlaps in resistant *E. coli* genotypes in human stool and household water, but lacked molecular resolution to confirm direct transmission [127]. A promising frontier involves integrating high-resolution, whole-genome sequencing with wastewater-based epidemiology and clinical surveillance, though such efforts remain logistically and economically constrained in many regions [128].

### 6.1. Wastewater Discharge and Effluent Release

Hospital wastewater is a significant reservoir of multidrug-resistant pathogens (e.g., *K. pneumoniae*, *P. aeruginosa*) and clinically relevant ARGs, such as carbapenemases (*bla*_KPC_, *bla*_NDM_) and ESBL (*bla*_CTX-M_). These effluents often contain high concentrations of antibiotics, disinfectants, and biocides, which exert selective pressure, enriching resistant strains [38,129]. Urban sewage systems collect antibiotics from human excretion, while industrial effluents (e.g., pharmaceutical manufacturing, livestock farms) introduce antibiotics, heavy metals, and ARGs. For example, tetracycline and sulfonamide residues are frequently detected in municipal wastewater, promoting ARG transfer via HGT [130]. When this partially treated water is discharged into rivers, lakes, or coastal waters, it acts as a direct input of resistance determinants into the natural environment [131]. These effluents may come from clinical settings where high antibiotic use selects for resistant strains, thereby enriching the environmental resistome [132].

### 6.2. Agricultural Runoff and Manure Application

The use of antibiotics in livestock (e.g., tetracyclines, macrolides) results in up to 90% of the administered drugs ending up in manure. When applied untreated to fields, manure introduces residual antibiotics, ARB, and ARGs into soils, which are subsequently dispersed via rainfall runoff or wind erosion [133,134]. ARGs like *ermB* (macrolide resistance) and *qnrS* (quinolone resistance) adhere to crops such as lettuce and spinach, posing foodborne transmission risks [133,135]. Birds and rodents grazing on manure-amended fields spread ARB (e.g., *E. faecalis* with *vanA*) to distant ecosystems [136]. Heavy metals in manure (e.g., zinc from feed additives) co-select for metal tolerance genes (*czcA*) and ARGs (*bla*_TEM_), amplifying resistance.

### 6.3. Sediments as Long-Term Reservoirs

Sediments in rivers, lakes, and coastal areas accumulate bacteria and organic matter over time. They act as long-term reservoirs for ARB and ARGs because the conditions in sediments (e.g., low oxygen, high nutrient content) support bacterial survival and even growth. These reservoirs can slowly release resistant bacteria into the water column, especially during disturbances like storms or human activity [53,137]. For example, *E. coli* with *bla*_CTX-M-15_ persists in river sediment biofilms despite seasonal fluctuations in antibiotic concentrations [103].

### 6.4. Soil–Water Interactions and Irrigation Return Flows

When treated or untreated wastewater is applied to agricultural fields or used for irrigation, ARGs and ARB infiltrate soils and leach into groundwater. In arid regions, irrigation return flows (IRFs) have been shown to carry multidrug-resistant *E. coli* and *Enterococcus* spp. into adjacent streams, creating a cyclical exchange between soil and surface water that amplifies environmental resistance burdens [82].

### 6.5. Wildlife-Mediated Dissemination

Wild birds, fish, and small mammals frequent multiple water bodies and can pick up ARB from one site (e.g., a polluted wetland) and deposit them in another (e.g., a pristine lake). For instance, migratory waterfowl have been found to carry class 1 integron-bearing bacteria in their feces, effectively linking disparate aquatic ecosystems in a single resistance network [136].

### 6.6. Aerosolization and Recreational Exposure

High-pressure water sprays in irrigation or desalination plants and wave action along coasts can generate aerosols laden with ARB and ARGs. Inhalation or contact with these bioaerosols represents an underappreciated transmission route, especially for occupational and recreational water users, and warrants targeted study of airborne resistance in coastal and agricultural settings [102].

These environmental pathways illustrate that aquatic ecosystems serve as interconnected reservoirs for AMR. Wastewater discharge, agricultural runoff, biofilm formation, sediment accumulation, microplastics, chemical stressors, and robust horizontal gene transfer contribute to ARB and ARGs’ persistence and spread in natural waters [138]. The interplay of these factors increases the risk of resistant infections via direct exposure (e.g., through drinking, recreation, or irrigation) and facilitates the integration of environmental resistance into human pathogens, posing a serious public health challenge [1].

Recent studies have shed light on how aquatic environments act as dynamic reservoirs and transmission pathways for AMR. Research indicates that antibiotic residues, heavy metals, and other pollutants discharged from municipal, hospital, and industrial sources persist in rivers, lakes, and even drinking water systems, creating selective pressures that favor ARB and facilitate horizontal gene transfer of ARGs [139,140].

For example, Xi et al. showed that although drinking water treatment processes reduce overall microbial loads, regrowth in the distribution system, often within biofilms, can lead to higher relative abundances of ARGs [46]. Similarly, studies by Chen et al. and Sanganyado & Gwenzi have documented that conventional disinfection methods (like chlorination) may not completely eradicate ARB; instead, they can sometimes select for resistant populations that persist in treated water [45,74].

Xu et al. employed high-throughput sequencing to profile ARGs in drinking water treatment plants and distribution networks. Their work showed that conventional processes, while reducing overall bacterial counts, may inadvertently select for resistant organisms [141]. Similarly, Lu et al. examined sand settling reservoirs. They found that ARGs associated with β-lactam and tetracycline resistance were consistently present, suggesting that natural sediments act as long-term reservoirs [142]. Moreover, Karkman et al. highlighted that fecal pollution from human and animal sources significantly contributes to the ARG abundance in surface waters, linking agricultural runoff and wastewater discharge to environmental AMR [143].

Additional work has assessed the role of emerging pollutants. For example, studies on microplastics [144] indicate that these particles provide a robust substrate for biofilm formation, thereby enhancing the horizontal transfer of ARGs among bacteria such as *E. coli* and *P. aeruginosa* [144]. Furthermore, investigations into heavy metal pollution [114] have shown that metals like mercury and arsenic co-select for antibiotic resistance, as resistance mechanisms are often genetically linked on mobile elements [114].

## 7. Mitigation Strategies and Future Directions

### 7.1. Wastewater-Based Epidemiology (WBE) as a Surveillance Tool

Over the past five years, wastewater-based epidemiology has emerged as a powerful approach for tracking viral outbreaks and monitoring antibiotic resistance trends in real time. By sequencing metagenomes from raw influent at key WWTPs, researchers can quantify hundreds of ARG families and correlate their abundance with antibiotic consumption data from pharmacy records [145]. For example, researchers used WBE in Barcelona to detect early rises in carbapenem resistance genes two weeks before hospital admissions increased, providing a critical window for public health intervention. Integrating WBE into national AMR action plans could offer continuous, community-level insights into emerging resistance hotspots and treatment failures [146].

### 7.2. Advanced Treatment Technologies

Advanced treatment technologies are increasingly critical for mitigating antibiotic resistance in wastewater effluents. Advanced oxidation processes (AOPs), including ozonation, UV/H_2_O_2_ oxidation, and Fenton reactions, generate non-selective hydroxyl radicals capable of degrading over 90% of common antibiotics such as tetracyclines and sulfonamides, while also reducing mobile genetic elements like class 1 integrons [147]. Pilot-scale systems coupling ozone with UV irradiation have shown enhanced micropollutant breakdown and a marked decrease in the regrowth potential of ARB [148]. Similarly, membrane bioreactors (MBRs), which combine biological treatment with microfiltration or ultrafiltration, can achieve 4–6 log reductions in ARB and substantial declines in antibiotic resistance gene (ARG) abundance when operated under optimized conditions [34]. However, biofilm formation on membrane surfaces can inadvertently enrich ARGs such as *bla*_NDM_ and *sul1*, necessitating rigorous cleaning protocols [78]. To overcome this, sequential MBR–AOP trains are deployed to ensure oxidative destruction of any ARGs surviving primary filtration [149]. In addition, adsorption onto granular activated carbon (GAC) effectively removes trace antibiotics and extracellular DNA fragments, reducing concentrations to nanogram-per-liter levels through pore diffusion and surface interactions. Recent research on engineered sorbents like functionalized biochars and metal–organic frameworks further demonstrates that tailoring surface chemistry enhances pollutant uptake and enables sorbent regeneration, offering a sustainable, low-energy option for polishing effluent before environmental discharge [149].

Looking forward, emerging technologies and transdisciplinary strategies hold promise in reshaping how we monitor, manage, and mitigate aquatic antibiotic resistance.

## 8. Future Directions

The escalating threat of antibiotic resistance in aquatic environments demands a paradigm shift toward innovative, interdisciplinary solutions. A critical priority lies in advancing material science to revolutionize water treatment technologies. Functionalized nanoparticles, graphene oxide membranes, and catalytic composites show promise in selectively degrading antibiotic residues and extracellular resistance genes, particularly for recalcitrant pollutants that evade conventional methods. Concurrently, integrating artificial intelligence with wastewater-based epidemiology could transform surveillance capabilities. Machine learning algorithms trained on metagenomic datasets from wastewater may predict emerging resistance trends, such as the spread of carbapenemase (*bla*_NDM_) or colistin (*mcr*-*1*) genes, enabling preemptive public health interventions. Equally urgent is elucidating the role of microplastics as vectors for resistance dissemination. Research must quantify how plastisphere biofilms facilitate horizontal gene transfer between environmental and pathogenic bacteria, particularly in marine ecosystems where microplastic pollution intersects with aquaculture and coastal urbanization.

While technological and policy advancements are essential, they must be contextualized within global socio-economic inequities. Many low- and middle-income countries (LMICs) lack infrastructure for centralized wastewater treatment and antimicrobial monitoring. In such settings, low-cost interventions are critical. Promising solutions include solar-driven advanced oxidation processes, constructed wetlands, gravity-fed sand or biochar filters, and natural coagulants, all of which have shown efficacy in reducing ARGs and antibiotic residues [149]. Additionally, international cooperation must support regulatory enforcement, sustainable pharmaceutical waste disposal, and capacity building for microbial surveillance. One Health frameworks should prioritize equitable access to these technologies and ensure that LMICs are not left behind in global AMR mitigation.

We acknowledge the limited representation of studies from North America, Latin America, and Oceania in this review. Future research should prioritize targeted surveillance in these underrepresented regions to build a more globally comprehensive understanding of aquatic antibiotic resistance. These regions often have distinct environmental pressures and antimicrobial usage profiles, yet remain poorly characterized. Coordinated international efforts and expanded surveillance initiatives aligned with WHO GLASS and UNEP are needed to close these geographic data gaps.

Additionally, international cooperation must support regulatory enforcement, sustainable pharmaceutical waste disposal, and capacity building for microbial surveillance. One Health frameworks should prioritize equitable access to these technologies and ensure that LMICs are not left behind in global AMR mitigation.

Longitudinal ecological assessments are needed to evaluate the cascading impacts of antibiotic resistance accumulation in aquatic systems. These studies should address how sediment-bound resistance genes alter microbial community dynamics, disrupt nutrient cycling, and propagate resistance to non-target species, including keystone microorganisms and aquatic fauna. Parallel efforts must address behavioral and regulatory gaps. Public education campaigns to curb antibiotic misuse in healthcare and agriculture should be reinforced by policies enforcing stricter pharmaceutical discharge limits and “polluter pays” principles for industries contributing to aquatic AMR. However, technological and policy advances alone will fail without addressing global inequities. Low-resource regions urgently require decentralized, cost-effective solutions, such as solar-driven advanced oxidation processes or biochar filtration systems, supported by international funding and technology transfer programs. Finally, a One Health framework is indispensable for harmonizing antibiotic usage data across human, veterinary, and environmental sectors.

## 9. Conclusions

As dynamic reservoirs of antibiotic resistance, water systems pose a formidable challenge to global health. This review underscores that wastewater discharges, agricultural runoff, and inadequate treatment processes perpetuate the environmental persistence of ARBs and ARGs. Key mechanisms—such as biofilm-mediated gene transfer, microplastic-facilitated dissemination, and co-selection by chemical stressors—amplify resistance spread across ecosystems. Clinically relevant pathogens like *E. coli*, *P. aeruginosa*, and *K. pneumoniae* persist in drinking water, irrigation sources, and recreational waters, directly threatening human health through exposure and foodborne transmission.

Current mitigation strategies, including advanced oxidation processes and membrane bioreactors, show promise but require optimization to address regrowth risks and infrastructure limitations. Furthermore, emerging pollutants such as microplastics and nanoparticles’ role in driving resistance remains poorly understood. A paradigm shift toward One Health approaches is imperative, integrating surveillance, policy, and technology to disrupt environmental transmission pathways. Without coordinated global action, the silent proliferation of resistance in water will undermine decades of medical progress. Safeguarding aquatic ecosystems is not merely an environmental imperative but a cornerstone of planetary health.

## Figures and Tables

**Table 2:** Comparative ARG patterns.

Water Source	Region	Top ARG Families	Detection Methods
WWTP effluents	Europe, Asia	*sul1*, *blaTEM*, *tetA*	qPCR, metagenomics
Agricultural runoff	North America, EU	*tetM*, *sul2*, *ermB*	culture, qPCR
Hospital effluents	North Africa, Asia	*blaNDM*, *mcr-1*, *intI1*	metagenomics
Recycled water	South America	*sul1*, *qnrS*, *blaCTX-M*	qPCR

## Data Availability

The data presented in this study are available within the article. Raw data supporting this study are available from the corresponding author upon reasonable request.
